# RosettaScripts: A Scripting Language Interface to the Rosetta Macromolecular Modeling Suite

**DOI:** 10.1371/journal.pone.0020161

**Published:** 2011-06-24

**Authors:** Sarel J. Fleishman, Andrew Leaver-Fay, Jacob E. Corn, Eva-Maria Strauch, Sagar D. Khare, Nobuyasu Koga, Justin Ashworth, Paul Murphy, Florian Richter, Gordon Lemmon, Jens Meiler, David Baker

**Affiliations:** 1 Department of Biochemistry, University of Washington, Seattle, Washington, United States of America; 2 Department of Biochemistry, University of North Carolina, Chapel Hill, North Carolina, United States of America; 3 Center for Structural Biology, Vanderbilt University, Nashville, Tennessee, United States of America; 4 Howard Hughes Medical Institute, University of Washington, Seattle, Washington, United States of America; University of South Florida College of Medicine, United States of America

## Abstract

Macromolecular modeling and design are increasingly useful in basic research, biotechnology, and teaching. However, the absence of a user-friendly modeling framework that provides access to a wide range of modeling capabilities is hampering the wider adoption of computational methods by non-experts. RosettaScripts is an XML-like language for specifying modeling tasks in the Rosetta framework. RosettaScripts provides access to protocol-level functionalities, such as rigid-body docking and sequence redesign, and allows fast testing and deployment of complex protocols without need for modifying or recompiling the underlying C++ code. We illustrate these capabilities with RosettaScripts protocols for the stabilization of proteins, the generation of computationally constrained libraries for experimental selection of higher-affinity binding proteins, loop remodeling, small-molecule ligand docking, design of ligand-binding proteins, and specificity redesign in DNA-binding proteins.

## Introduction

The Rosetta software suite for macromolecular modeling has useful applications in many areas of interest to molecular biologists. It allows the redesign of protein structure[1] and has been used to generate new protein folds[2], stabilize enzymes[3], generate novel enzymes[4], [5], protein-protein interactions and inhibitors[6], and redesign specificities in protein-protein[7] and protein-DNA interactions[8]. The design of new or improved protein function often requires detailed treatment of available degrees of freedom, typically on a case-by-case basis. Such case-specific properties favor a user interface that is flexible enough to allow control of individual degrees of freedom and the course of the modeling trajectory. Additionally making the modeling approaches developed in Rosetta available to the wide community of molecular biologists, with varying proficiencies in programming, demands a framework that does not suffer from the rigidities of traditional programming languages.

With these goals in mind, we developed RosettaScripts, an XML-like language for specifying modeling protocols in the Rosetta framework (specification of the XML format can be found at http://www.w3.org/TR/2000/REC-xml-20001006). RosettaScripts provides protocol-level access to modeling functionalities, such as loop modeling, rigid-body docking, and sequence design. Protocols can be dovetailed to generate complex trajectories comprising, for instance, a phase of low-resolution rigid-body docking, followed by filtering according to residue-specific contacts, sequence redesign of parts of an interface, and finally all-atom docking and minimization. The protocols can be written quickly, do not require recompilation of the Rosetta C++ source code, and can be ported and executed on all computing platforms that support Rosetta, thus opening the door to fast development and testing for non-experts.

In this paper we describe how to use RosettaScripts, providing concrete, working examples for a variety of modeling tasks. Detailed usage instructions of each of the RosettaScripts functionalities are available at the RosettaCommons website (http://www.rosettacommons.org) and are updated with each public release of the source code. The programming section below explains how the RosettaScripts framework was implemented within Rosetta as well as the logic for extending RosettaScripts with new functionalities.

## Results

**Figure 1:**
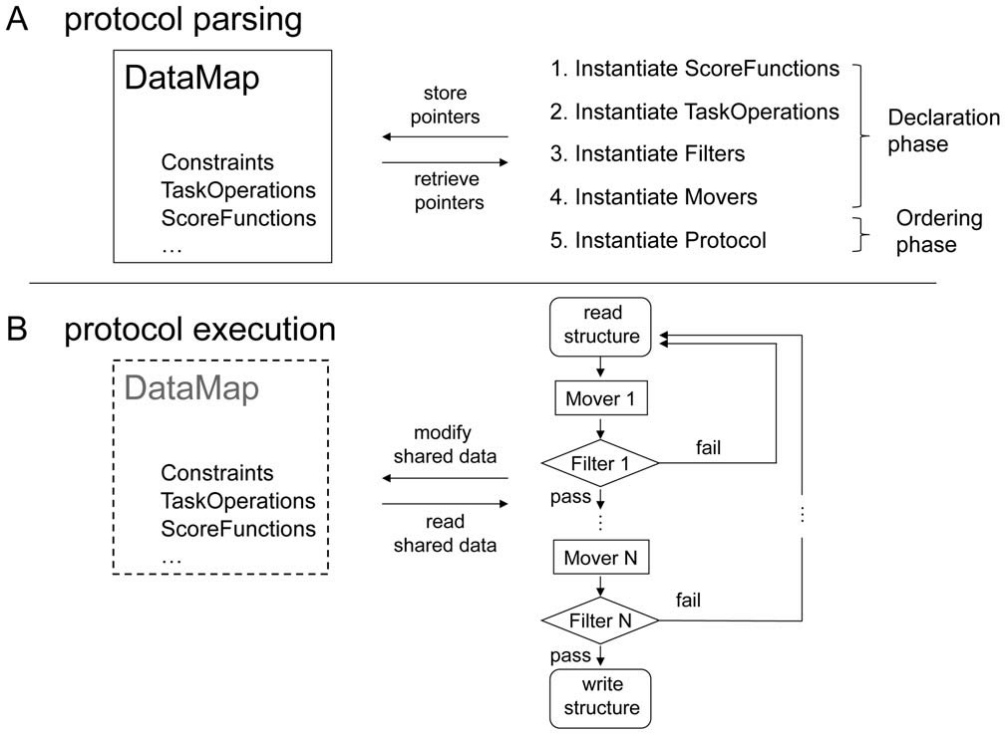
A schematic of RosettaScript operations. (A) When parsing an XML protocol, a series of objects are instantiated. The DataMap is used to store some of these elements as they are parsed, and to store any additional objects that the elements define (e.g., constraints from text files). Movers and Filters can access and modify the elements stored in the DataMap. After parsing completes, the DataMap is deallocated, though the objects it once held may persist in memory. (B) Starting from a structure read in from disk, protocol execution consists of a series of Mover and Filter applications. A structure can either pass or fail a Filter: failure causes execution to return to the beginning, whereas success causes execution to proceed. At the end of execution a protein model and its score are written to file. Though the DataMap does not persist beyond the parsing of the XML file, any of its former elements that are pointed to by Movers and Filters remain in memory, thus allowing communication between Movers and Filters during execution.

RosettaScripts relies on the object-oriented architecture of Rosetta 3.0. A detailed description of the Rosetta 3.0 programming framework is available in ref. [9]. At the most general level, a script consists of a declaration phase and an ordering phase – it reads like a recipe starting with an ingredient list and finishing with a sequence of steps (Figure 1). In the declaration phase, the user declares a set of Movers (objects to modify a structure), Filters (objects to evaluate a structure), ScoreFunctions (objects to evaluate the energy of a structure), and TaskOperations (objects to control how Rosetta's side-chain placement routines, “the packer,” should operate). In the ordering phase, the user lays out the steps of the protocol by stating the order in which the Movers and Filters should be applied. Step 1 is always the same and is handled by the JobDistributor (described later): a structure is read in from disk (or elsewhere); steps 2 through *n* describe how Rosetta should modify that structure. If a filter is applied at step *i,* and the structure fails the filter, then execution returns to step 1 for another attempt. Finally, if a structure passes all filters, it is returned to the JobDistributor for output to disk.

Mover objects modify a structure (henceforth, a Pose) in some way. The vast majority of Rosetta routines for modifying the structure of a protein are wrapped within Movers, from the simplest steps, such as minimizing, to more complicated protocols such as rigid-body docking. Complex protocols are often implemented as Movers built from a series of simpler Movers. Filter objects evaluate a Pose in their “apply” function and return a boolean describing whether or not the structure passed the filter. Filters are useful in aborting trajectories that are headed towards uninteresting regions of conformation and sequence space. Both Movers and Filters often require a substantial amount of data to control their behavior precisely. In Rosetta2, developers were only able to tune their protocols with command-line flags. In an object-oriented framework, where a programmer can have multiple instances of the same Mover, the command line is overly limited, preventing the user from expressing nuance because a single flag cannot carry two meanings: e.g., “use a cutoff of 10 in step 3, use a cutoff of 20 in step 8.” RosettaScripts remedies this problem by letting the user specify data for controlling Movers and Filters in XML-like blocks, where each block can carry different pieces of data.

### Parsing a RosettaScript

A RosettaScript is parsed as a series of XML-like entries called Tags. A Tag is a recursive data structure, each instance of which contains two mappings: from strings to strings (i.e., options), and from strings to Tags (subtags); the use of Tags is described in greater detail below. At the highest level, a RosettaScript consists of five tags, each of which contains subtags: MOVERS, FILTERS, SCOREFXNS, TASKOPERATIONS, and PROTOCOLS (Fig. 1A). The only tags that must be specified are MOVERS and PROTOCOLS. Information specified within each of the first four high-level Tags is explained above; the PROTOCOLS section defines the order of Movers and Filters to be executed for each trajectory. The following provides a skeleton script:

<ROSETTASCRIPTS>

<SCOREFXNS>

</SCOREFXNS>

<TASKOPERATIONS>

</TASKOPERATIONS>

<FILTERS>

</FILTERS>

<MOVERS>

</MOVERS>

<PROTOCOLS>

</PROTOCOLS>

</ROSETTASCRIPTS>

where the high-level tags would be populated with concrete objects prior to execution. Strings outside of the <> brackets are treated as comments and ignored by the RosettaScripts machinery.

### TaskOperations

Many of the discrete conformational sampling capabilities of Rosetta are implemented using a rapid Metropolis-Monte Carlo simulated annealing engine referred to as the packer[9]. The packer searches for low-energy combinations of side-chain conformations. TaskOperations define the residue conformations and identities used in the search and can be used to focus design or prediction to the relevant spaces (e.g., to design only interfacial residues and repack the remainder).

From a technical standpoint, TaskOperations are used to constrain a PackerTask class. PackerTasks are one-time-use objects and are constructed immediately before use. They are constructed by the TaskFactory class, and modified by TaskOperations before being handed to the packer. The PackerTask is peculiar in that all of its operations are commutative – the final state of the PackerTask is independent of the order in which TaskOperations are applied to it. The analogy of refining the PackerTask is to sculpting a block of marble: the task starts out with the command “redesign all residues allowing all amino acids” and successive operations are allowed to restrict the focus of the task to fewer residues or fewer amino acids at particular residues. However, once rock is chipped off, it cannot be put back; once a residue is disabled or an amino acid is marked as unavailable, it cannot be re-enabled. TaskOperations restrict the degrees of freedom of PackerTasks. The advantages of this system (that the state of the PackerTask is independent of the order of operations, removing all possible ambiguity about its state and avoiding any issues of operation precedence) are somewhat offset by the disadvantage that TaskOperations sometimes step on each other's toes: one TaskOperation will often disable a residue that another TaskOperation would say should be designable.

There are three general types of TaskOperations: 1) those without options: a general, uniform behavior is applied over the whole Pose; 2) those with options: parameter-dependent behavior(s) are applied to the whole Pose; and 3) residue-level TaskOperations: behavior is applied to a subset of Pose residues that belong to a certain category. The following is an excerpt from a script providing examples for all three usages:

…

<TASKOPERATIONS>

<InitializeFromCommandline name = ifcm/> to make the Packer aware of command-line options

<RestrictToRepacking name = no_mutations/> only repack; do not design

<ReadResfile name = rrf filename = myresfile/> resfiles are external files that describe which residues are allowed to pack

<OperateOnCertainResidues name = fix20to24>

<PreventRepackingRLT/> do not change residues

<ResidueIndexIs indices = 20,21,22,23,24/>

</OperateOnCertainResidues>

<OperateOnCertainResidues name = keepNonpolars>

<RestrictToRepackingRLT/> only repack; do not design

<ResidueLacksProperty property = POLAR/> operate on polar residues

</OperateOnCertainResidues>

</TASKOPERATIONS>

…

<MOVERS>

<PackRotamersMover name = packer scorefxn = score12 task_operations = ifcm,no_mutations/> score12 is the default all-atom Rosetta energy function; PackRotamersMover invokes the Packer to design or repack sidechains

<PackRotamersMover name = resfilepacker scorefxn = score12 task_operations = ifcm,rrf/>

<PackRotamersMover name = anotherpacker scorefxn = score12 task_operations = ifcm,fix20to24,keepNonpolars/>

</MOVERS>

…

In this excerpt, three PackRotamersMovers will be instantiated with different functionalities, since each one relies on a different set of TaskOperations.

### Tests that ensure source-code integrity

The Rosetta source code includes several software tests that demonstrate and ensure the proper functioning of the RosettaScripts platform. The “integration” tests (e.g., test/integration/tests/dna_interface_design) are fully featured demonstrations of established protocols. Additional tests of RosettaScripts functionality include hotspot_graft, place_simultaneously, rosetta_scripts_setup, ligand_dock_script, rotamer_recovery, and score12_docking. In addition, the “scientific” tests (test/scientific/) carry out full benchmark analyses of common modeling procedures. This includes an additional dna_interface_design test to compute the mutational recovery rates for native amino acids and nucleotides across a diverse set of 72 sequence-specific protein-DNA interfaces, as well as a ligand_docking benchmark based on 20 crystal structures. Current and past results of these tests are available on the RosettaTests Server (http://rosettatests.graylab.jhu.edu/tests).

### Programming Details

#### Tag class

The Tag class implements a recursive-descent parser that translates a text document to an object-oriented, template-based data structure representing the contents of that document. The production rules for the language in Extended Backus–Naur Form (EBNF) are:

Tag: =  < Name Option* > Tag* </Name > | <Name Option*/>

Option: =  Name  =  Value

Name: =  (string without whitespace)

Value: =  (string without whitespace) | “ (string with whitespace) ”

These production rules are intended to bear resemblance to the HTML (http://www.w3.org/TR/REC-html32) or XML (http://www.w3.org/XML/) languages, which are simple to learn and familiar to many users of Rosetta. Full-fledged XML parsers were not used since no text was to be annotated, other than the structure of the Tags themselves. Also, since verification of the semantic structure of the tags is left entirely to the user for the sake of simplicity, the formal document verification abilities of XML were not required. The parser is implemented with the Boost Spirit library (http://boost-spirit.com/home/).

The Tag class itself allows recursive access to its subtags via the getTag and getTags functions. It also allows access to values of the options through the templated getOption<> function, which, for convenience, returns its data as any type for which operator>>(string,T) is defined. Implementation of this function allows users to further define the behavior of the Tag class without changes to the underlying parser.

#### Parsing Tags

The parsing of individual Tags is handled similarly throughout the various sections of a RosettaScript. For each section, a factory is relied upon that indirectly maps a set of unique hardcoded names (e.g., “PackRotamersMover”) to a set of objects (e.g., the PackRotamersMover). When a Tag is read, the relevant factory queries its creators for one that will build an object with the same name as the Tag's name (factories and creators are described in greater detail below). If the factory finds such a creator, it then requests an instance of the corresponding object and calls that object's parse_my_tag routine with the options specified within the Tag. The parse_my_tag routine interprets the information provided by the user and saves that information in internal variables within the object, e.g., cutoffs that would control the object's behavior. The instantiated object is saved in a map for subsequent use in the ordering phase: Mover objects are saved in a Movers map; Filter objects are saved in a Filters map.

Most top-level Tag types require an option called “name” (Movers, Filters, TaskOperations, but not ScoreFunctions). This name identifies different instances of the same class so that they can be referred to separately in the ordering section. For example, in the MOVERS section, the following Tag, <PackRotamersMover name = design task_operations = design_shell/> would provoke the MoverFactory to create a new instance of the PackRotamersMover, and call this instance's parse_my_tag() routine with the option task_operations = design_shell. This Mover would be saved in the Movers map under the name “design” for use in the ordering phase. Note that this mover is handed the name “design_shell” for the TaskOperation it should use; the Mover will look for this TaskOperation in a data structure called the DataMap (discussed in greater detail in the next section).

Once all of the declarations have been parsed, we arrive at the ordering section (labeled PROTOCOLS in a RosettaScript). Here, a Mover called ParsedProtocol is instantiated and an order-dependent array of Movers and Filters is instantiated with it, explicating the sequence of Movers and Filters to be executed in a trajectory (Fig. 1B). Consider for example, the following section:

…

<PROTOCOLS>

<Add mover = design filter = number_of_contacts/>

<Add mover = dock/>

</PROTOCOLS>

…

RosettaScripts would query the Movers map for Movers, the name option of which was design and dock and the Filters map for the Filter number_of_contacts, all of which would be declared in previous sections. ParsedProtocol's internal array of Movers and Filters will have the sequence design → number_of_contacts → dock, which will be executed in this order at run-time. As demonstrated in several applications below, ParsedProtocol itself is a Mover that can be declared in the Movers section to aggregate a sequence of Movers under one name. For example,

<ParsedProtocol name = aggregate_mover>

<Add mover = design filter = number_of_contacts/>

<Add mover = dock/>

</ParsedProtocol>

The Mover aggregate_mover can then be called in the PROTOCOLS section.

Once a script has been parsed, the Movers map, the Filters map, and the DataMap all leave scope, and a smart pointer is retained in memory to the ParsedProtocol mover from the ordering phase (Fig. 1B). Any objects held in these maps that are not pointed to from the ParsedProtocol mover (or from an object contained in the ParsedProtocol mover) will be automatically deleted.

#### The DataMap

Global variables are discouraged in large software projects[10], so a specialized mechanism for communicating information between Movers and Filters is needed. To this end, we introduced a DataMap object (Fig. 1). The DataMap is a map from strings to maps: for example, the “ScoreFunctions” string points to a map with keys such as “score12” and “score_docking”, each of which points to smart pointers of the relevant ScoreFunctions. (Fig. 1A). This flexible prototype allows any object type to be defined within this framework, for instance, both TaskOperations and ScoreFunctions are held in this map.

The DataMap is passed along with the Movers and Filters maps to the individual objects at parse time (Fig. 1A). It provides a templated accessor function for data retrieval, which carries out a type-safety check (using C++'s dynamic_cast function) and returns a pointer to the requested object. For example, the code data_map.get< ScoreFunction * >(“scorefxns”, “high_resolution”) would request a pointer to ScoreFunction with the two defining strings “scorefxns” (the name of the map of strings to ScoreFunction pointers) and “high_resolution” (the name of a particular ScoreFunction). The DataMap can also be used to communicate information between Movers and Filters during run-time (Fig. 1B). For instance, one Mover might instantiate and store a ScoreFunction object in the DataMap that is then accessed and modified by other Movers or Filters. Since these Movers would all hold pointers to the same ScoreFunction, the weights on individual score terms for a single ScoreFunction instance could be modified in the course of a trajectory, and this modification would affect all the Movers using that instance.

#### The Job Distributor

The JobDistributor is a Rosetta 3.0 framework, the main task of which is central handling of structure and scoring input/output operations. RosettaScripts execution is embedded within the JobDistributor and can therefore access its structure-reading and -writing functionalities. Options for controlling JobDistributor behavior[9] are set through the command line. For instance, reading of a Protein Databank (PDB) file is accessed through the command line -in:file:s, whereas reading of a more compact form of a coordinates file is specified by -in:file:silent[9]. Similarly, coordinate files can be written as PDB or silent files with the corresponding command line options.

### Extending RosettaScripts to include new Movers and Filters

The XML-parser in RosettaScripts relies on factory classes to instantiate Movers and Filters by name, without having to know any details about particular Movers or Filters. This means that minimal effort is required to include new objects into the RosettaScripts API. A new Mover merely has to register itself with the MoverFactory before the parsing step asks the factory for an instance of that Mover. This means that new Movers can be added a) easily by new Rosetta developers by adding a handful of classes and modifying one existing source file; b) easily by non-Rosetta programmers who are linking against the Rosetta3 libraries; or c) easily in an interactive Python session, or from a Python script using the PyRosetta libraries[11]. In this last case, the new Mover being introduced can be a Python-defined subclass of the C++ Mover base class.

**Figure 2:**
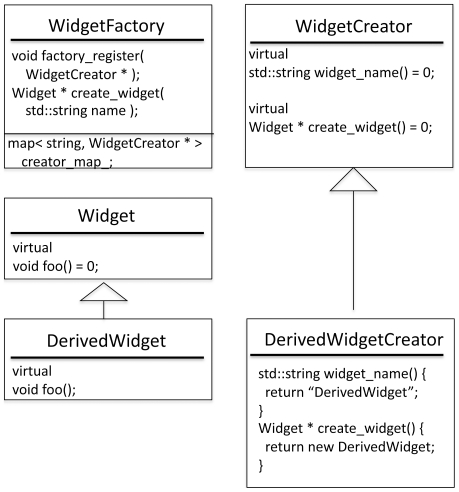
Unified-Modeling Language (UML) class diagram of Rosetta's factory scheme. The creation of Movers, Filters, and TaskOperations is controlled by similar factory setups. Pictured here are the classes responsible for the instantiation of generic “Widget” classes. The singleton WidgetFactory maintains a map from strings to WidgetCreators. Each WidgetCreator is responsible for instantiating a particular Widget; e.g., the derived WidgetCreator class, DerivedWidgetCreator, is responsible for instantiating the derived Widget, DerivedWidget. The factory registration system allows new Movers, Filters, and TaskOperations (and their corresponding MoverCreators, FilterCreators, and TaskOperationCreators) to be defined outside of the Rosetta3 libraries and yet to be included in the RosettaScripts framework without requiring the addition of any new dependencies to the Rosetta3 libraries.

The factory scheme is made up of four players: class Widget, class WidgetFactory, class WidgetCreator, and class WidgetRegistrator (Fig. 2). Class WidgetFactory is a singleton[10] that holds a map from strings to WidgetCreators. Each WidgetCreator is responsible for instantiating a particular class derived from class Widget and for reporting a unique name for instances of this derived Widget class. WidgetCreators must be registered with the WidgetFactory, at which time the WidgetFactory adds the WidgetCreator to its map of strings WidgetCreators. The WidgetFactory defines a function: Widget * create_widget(std::string const & name) wherein it searches for a WidgetCreator with the given name and then asks that WidgetCreator for a new Widget. The WidgetRegistrator objects, at construction, create a particular WidgetCreator and register it with a particular WidgetFactory. The registrators are placed in the library init.cc files (e.g., src/protocols/init.cc) which also house an init() function that all Rosetta executables must call at the very beginning of main(). This ensures that registration happens before the factories are used. New Widgets and WidgetCreators can be defined anywhere, including in external libraries, and can be included in the RosettaScripts without modifying any of the code in the Rosetta3 libraries. This flexibility would not be possible without separating the role that WidgetCreators play (instantiating particular Widgets) from the role the WidgetFactory plays (centralizing the instantiation).

### Applications

In the following, all scripts can be run using the RosettaScripts application that is part of the Rosetta release with the following commandline:

rosetta_scripts –s <PDB file name> -parser:protocol <XML file name> -database <location of the Rosetta database>.

For convenience, we provide input and output files for each protocol as Supplemental Information S1.

#### Flexible backbone design for monomers

Protein cores are usually very well packed with mostly hydrophobic amino acids, to the extent that the packing density corresponds to a close-packed crystal[12]. Cavity-forming mutations or strain in the core of natural proteins compromise their stability[13]. Therefore, generating stable monomeric proteins demands a tightly packed and strain-free core[3]. In addition, numerous studies have pointed out that native proteins evolved by optimizing their backbone conformations in the process tightening core packing(e.g., [1]). This idea directly led to the development of a design protocol for sequence and backbone optimization and the generation of the first computationally designed novel protein fold[2]. Accordingly, the flexible-backbone design protocol implemented here provides the simultaneous design of sequence and backbone (FlxbbDesign), followed by the filtering of the designed structures by RosetttaHoles (PackStat) [14], which quantitatively assesses protein packing:

<ROSETTASCRIPTS>

<SCOREFXNS>

<SFXN1 weights = score12_w_corrections>

<Reweight scoretype = atom_pair_constraint weight = 1.0/>

</SFXN1>

</SCOREFXNS>

<TASKOPERATIONS>

<LayerDesign name = layer layer = core_boundary_surface/>

</TASKOPERATIONS>

<FILTERS>

<PackStat name = pstat threshold = 0.60/>

</FILTERS>

<MOVERS>

<FlxbbDesign name = flxbb ncycles = 3 constraints_sheet = 100.0 sfxn_design = SFXN1 sfxn_relax = SFXN1 clear_all_residues = 1 task_operations = layer/>

</MOVERS>

<PROTOCOLS>

<Add mover = flxbb/>

<Add filter = pstat/>

</PROTOCOLS>

</ROSETTASCRIPTS>

In this example, FlxbbDesign performs 3 cycles of sequence design and backbone optimization[15]. Internally, initial sequence information is cleared by turning all residues to alanine (clear_all_residues = 1). Then, sequence design is conducted with the TaskOperation of LayerDesign, which specifies the allowed amino acid types depending on the extent of burial of each position (e.g., buried positions are allowed to be hydrophobic, whereas exposed positions are polar). Next, backbone optimization is performed with sheet constraints to prevent drastic changes to the tertiary structure. Finally, the extent to which the core of the structure is well-packed is evaluated and filtered by RosettaHoles[14] using the PackStat filter.

#### Protein-protein interface redesign and conformational sampling

Recent advances in protein engineering have allowed screening and selection of large-scale combinatorial protein libraries leading to novel therapeutics and biotechnological reagents[16]. Despite these advances the sequence space that can be sampled even by the most sophisticated experimental methods (10^11^ combinations) would allow the full randomization of a mere 8 independent amino acid positions. By contrast a typical protein-protein interface consists of more than 20 amino acid residues within the first shell of interactions, well beyond the reach of screening capabilities. Efficient computational protocols can readily sample conformational spaces of this size[17]. Computational design can therefore be useful in restricting sequence space to energetically reasonable identities and experimentally manageable sizes[18]. Here, we describe an approach to identify diverse sequence mutations in a protein interface that are compatible with a given binding mode. The protocol makes use of rigid-body docking, backbone and side-chain minimization and packing, sequence design, and a combination of score functions that encourages greater sequence diversity. Often, certain structural and energetic properties should be retained throughout a trajectory, e.g., the computed binding energy (ddG), the presence of particular hydrogen bonds or the root-mean-square deviation from the starting structure. The versatility of RosettaScripts allows the user to combine a set of movers and filters to ensure that these properties are maintained. The example protocol below starts with high resolution docking with soft-repulsive penalties. It uses iterations over a series of design movers combined with subtle backbone sampling defined through the Backrub mover[19], [20].

<ROSETTASCRIPTS>

<FILTERS>

<Ddg name = ddG scorefxn = score12 threshold = -15 repeats = 2/> binding energy calculation; an average of two repeats is computed for better numerical accuracy

<Sasa name = sasa threshold = 800/> Buried surface area upon complex formation

<Rmsd name = rmsd confidence = 0/> confidence = 0 means that the filter will be evaluated but not used as an acceptance criterion

<CompoundStatement name = ddg_sasa> combine filters into a single logical statement

<AND filter_name = ddG/>

<AND filter_name = sasa/>

</CompoundStatement>

</FILTERS>

<MOVERS>

<Docking name = docking score_high = soft_rep fullatom = 1 local_refine = 1/> Invokes RosettaDock local-refinement (in full-atom) with a soft potential

<Backrub name = backrub partner1 = 0 partner2 = 1 interface_distance_cutoff = 8.0 moves = 1000 sc_move_probability = 0.25 scorefxn = score12

small_move_probability = 0.15 bbg_move_probability = 0.25/> perturb the backbone of chain2

<RepackMinimize name = des1 scorefxn_repack = soft_rep scorefxn_minimize = soft_rep minimize_bb = 0 minimize_rb = 1/>

<RepackMinimize name = des2 scorefxn_repack = score12 scorefxn_minimize = score12 minimize_bb = 0 minimize_rb = 1/> Design & minimizatio

n at the interface

<RepackMinimize name = des3 minimize_bb = 1/>

<ParsedProtocol name = design>

<Add mover_name = des1/>

<Add mover_name = des2/>

<Add mover_name = des3/>

<Add mover_name = backrub/>

<Add mover_name = des3 filter_name = ddg_sasa/>

</ParsedProtocol>

<GenericMonteCarlo name = iterate scorefxn_name = score12 mover_name = design trials = 10/>

</MOVERS>

<PROTOCOLS>

<Add mover = docking/>

<Add mover = iterate/>

<Add filter = ddG/>

<Add filter = sasa/>

<Add filter = rmsd/>

</PROTOCOLS>

</ROSETTASCRIPTS>

In each trajectory, this protocol would carry out one docking step and then iterate 10 times (through use of the GenericMonteCarlo mover) over a set of design and backbone sampling (Backrub) steps. The output will include the modified protein structure, the computed binding energy (ddG), the buried surface area upon complex formation (sasa), and the RMSD from the starting structure (rmsd). Typically, several thousand models should be executed with this script and the mutations at the interfaces combined to constrain sequence space for experimental libraries.

#### Loop modeling

Polypeptide stretches lacking secondary structure are often found at protein interfaces. A well-known example is that of antibodies, which use a combination of loops to achieve high shape complementarity and interaction density to antigens with a wide variety of surface features. The variety of conformational solutions that loops provide suggests that incorporating loop modeling in the design of interfaces could substantially increase the potential utility of protein design. The example below provides a procedure for sampling conformational plasticity at the interface for use in increasing the affinity of a target-scaffold pair of proteins.

Alternative rigid-body orientations of a scaffold-target pair potentially present different loops to the target surface. We developed a customizable framework for the automatic detection of loops at the interface to be used for conformational remodeling and sequence design. These methods are optimized for use with interfacial loops, but can also be extended to incorporate backbone moves in the context of a monomer. First, the user specifies a LoopFinder Mover, with options available to define minimum and maximum loop lengths. The user may also specify whether loops should be restricted to the interface, and on which partners they should be detected. A subsequently invoked LoopRemodel Mover carries out the actual conformational remodeling and sequence design in the loops identified by the LoopFinder. Alternatively, if the loop spans to be remodeled are known ahead of time, the user may specify them within LoopRemodel itself. The LoopRemodel mover can perform both extensive loop building, in which backbone degrees of freedom are randomized prior to building, or refinement of an existing loop structure. Building operates in low-resolution mode, and so exclusively operates on backbone torsion angles. A refinement step operates in all-atom mode and intersperses backbone moves with side-chain repacking and/or design. A single LoopRemodel Mover can sequentially incorporate both building and refinement. Two loop-remodeling strategies are currently supported: fragment insertion[21] followed by cyclic coordinate descent[22], and kinematic loop closure[23], which vary in how torsion-angle sampling is conducted.

In many protein-protein interactions, the structure of each complexed partner differs somewhat from its *apo* structure. The aforementioned small backbone moves and loop remodeling can be linked together within RosettaScripts to approximate such interfacial conformation changes during the design process. This protocol uses a special wrapping mover called LoopOver, which allows a user to repeat combined sub-movers. The contained sub-mover is repeated until one of two exit conditions is fulfilled: either a certain number of repeats has occurred, or a user-defined Filter has passed. The script assumes a reasonable starting rigid-body orientation between two partners, with chain 1 being the target and chain 2 being the design scaffold. The script begins with large scale interfacial loop building of chain 2 in low-resolution mode, then proceeds to refine these new loop conformations with a soft-repulsive score function. Once the loop conformations have been refined with a fixed sequence, they are refined again with simultaneous sequence design and a more physically realistic score function (with higher repulsive weights). Repeated Backrub moves then subtly sample backbone conformations, and a set of three RepackMinimize moves is used for interface design. Target-specific filters may be added to this script to ensure that conformational and sequence space is constrained by known functional or energetic thresholds.

<ROSETTASCRIPTS>

<MOVERS>

<LoopFinder name = find ch1 = 0 ch2 = 1 interface = 1 min_length = 3 max_length = 10 mingap = 2/> Find loops on chain 2 with length 2>x>11, with at least 3 residue separation.

<LoopRemodel name = build auto_loops = 1 design = 0 protocol = kinematic perturb = 1 refine = 0/> Aggressively build found loops, no design

<LoopRemodel name = refine1 auto_loops = 1 design = 0 protocol = kinematic perturb = 0 refine = 1 refine_score = soft_rep/> Refine found loops, soft repulsive, no design

<LoopRemodel name = refine2 auto_loops = 1 design = 1 protocol = kinematic perturb = 0 refine = 1 refine_score = score12/> Refine found loops, score12, design

<Backrub name = backrub partner1 = 0 partner2 = 1/> Subtle backbone moves over whole interface of chain 2

<LoopOver name = repeat_backrub mover_name = backrub iterations = 10/> Bundles Backrub into repeats (no filter used)

<RepackMinimize name = des1 scorefxn_repack = score_docking scorefxn_minimize = soft_rep minimize_bb = 0 minimize_rb = 0/>

Aggressive design scorefunction, sidechain only moves

<RepackMinimize name = des2 scorefxn_repack = soft_rep scorefxn_minimize = score12 minimize_bb = 0 minimize_rb = 1/> More

constrained design scorefunction, sidechain and rigid body moves

<RepackMinimize name = des3 design_partner1 = 1 design_partner2 = 1 minimize_bb = 1/> Strict design scorefunction, sidec

hain, rigid body, and backbone moves

<ParsedProtocol name = design>

<Add mover_name = des1/>

<Add mover_name = des2/>

<Add mover_name = des3/>

</ParsedProtocol>

</MOVERS>

<PROTOCOLS>

<Add mover = find/>

<Add mover = build/>

<Add mover = refine1/>

<Add mover = refine2/>

<Add mover = repeat_backrub/>

<Add mover = design/>

</PROTOCOLS>

</ROSETTASCRIPTS>

#### Enzyme and ligand-binder design

RosettaScripts can be used for the modeling and design of protein-ligand interfaces as performed in the *de novo* computational design of enzymes[4], [5]. In the Rosetta enzyme-design methodology, the active-site geometry is specified by a set of pairwise geometric constraints – called match constraints[24] – each defined between the transition-state (TS) model and a functional group from the protein (catalytic residue). Starting from a given orientation of a ligand or a TS model with respect to a protein structure, match constraints between specified sidechains or backbones of catalytic residues and the TS can be applied and modulated during the simulation. The conformations and identities of the interface side chains, the rigid-body orientation of the TS with respect to the protein, and the conformation of an internally flexible TS model can be simultaneously optimized using a combination of Monte Carlo optimization and gradient-based minimization, with or without match constraints.

The AddOrRemoveMatchCsts Mover handles the application or removal of match constraints that are used to specify the relative orientation of the TS and a given functional group. Constraints can also be defined between two functional groups within the protein (e.g., a His-Asp dyad). Each instance of the Mover can take a different set of constraints, specified *via* a user-defined input file, thereby allowing the user to change the magnitude and/or the number of constraints used during the simulation trajectory. In cases where the TS model contains a covalently bound intermediate (e.g., acylenzyme intermediates), it is possible to specify retaining the covalent constraint even while removing other non-covalent ones.

The EnzRepackMinimize Mover performs Monte Carlo optimization of the identities and conformations of a protein-ligand interface followed by gradient-based minimization of the energy of the resulting interface. The interface is identified using the DetectProteinLigandInterface TaskOperation (see below). The user can toggle the minimization of various degrees of freedom – side chain, backbone, ligand rigid-body orientation, and the ligand internal torsion angles – individually during each instantiation of the mover, thus allowing fine-grained control over the degrees of freedom minimized at a particular stage of the protocol. If the option cst_opt is selected, all protein residues on the interface except catalytic residues are temporarily converted to alanines, and the energy including constraint energy – defined as a harmonic penalty from the ideal values – is minimized.

The DetectProteinLigandInterface TaskOperation determines the residue positions on a protein-ligand interface for subsequent protein design. This TaskOperation identifies ligand-proximal positions on the protein (or a user-specified set of residues) and marks them as designable; positions distal from the ligand are marked as immutable; and intermediate positions are marked repackable. During the course of an enzyme-design calculation, designable positions are allowed to change both their identity and conformation; repackable positions are allowed to change only their conformation; whereas immutable positions are constrained to their starting conformation. The following is a typical script for conducting an enzyme-design calculation.

<ROSETTASCRIPTS>

<SCOREFXNS>

<myscore weights = enzdes.wts/> Read the enzdes.wts score function from the Rosetta database

</SCOREFXNS>

<FILTERS>

<EnzScore name="allcst" score_type = cstE scorefxn = myscore whole_pose = 1 energy_cutoff = 5/> filter on the constraint scores

<LigInterfaceEnergy name="interfE" scorefxn = myscore energy_cutoff = -9.0/> filter on the energy across the interface

<CompoundStatement name="myfilter">

<AND filter_name="allcst"/>

<AND filter_name="interfE"/>

</CompoundStatement>

</FILTERS>

<MOVERS>

<AddOrRemoveMatchCsts name = cstadd cst_instruction = add_new/> add catalytic constraints

<EnzRepackMinimize name = cstopt cst_opt = 1 minimize_rb = 1 minimize_sc = 1 minimize_bb = 1/> optimize constraints energy in polyAla background

<EnzRepackMinimize name = desmin design = 1 repack_only = 0 scorefxn_minimize = myscore scorefxn_repack = myscore minimize_rb = 1 minimize_sc = 1 minimize_bb = 1 cycles = 1/>

<EnzRepackMinimize name = fin_min repack_only = 0 design = 0 scorefxn_minimize = myscore scorefxn_repack = myscore minimize_rb = 1 minimize_sc = 1 minimize_bb = 1 cycles = 1/>

<EnzRepackMinimize name = fin_rpkmin repack_only = 1 design = 0 scorefxn_minimize = myscore scorefxn_repack = myscore minimize_rb = 1 minimize_sc = 1 minimize_bb = 1 cycles = 1/>

<AddOrRemoveMatchCsts name = cstrem cst_instruction = remove keep_covalent = 1/> remove constraints

<AddOrRemoveMatchCsts name = cstfinadd cst_instruction = add_pregenerated/> add the last set of constraints added just prior to removing them (used for scoring typically at the end of the trajectory)

</MOVERS>

<PROTOCOLS>

<Add mover_name = cstadd/>

<Add mover_name = cstopt/>

<Add mover_name = desmin/>

<Add mover_name = cstrem/>

<Add mover_name = fin_min/>

<Add mover_name = fin_rpkmin/>

<Add mover_name = cstfinadd/>

</PROTOCOLS>

</ROSETTASCRIPTS>

#### Ligand docking and design

RosettaLigand allows the simultaneous sampling of protein, ligand, and rigid-body degrees of freedom[25], [26] and has been refactored for use with RosettaScripts. Separating the protocol into a collection of scriptable movers allows users to customize the docking study in fine detail. This opens the door to novel ligand-docking approaches while preserving the benchmark results seen previously. Multiple ligands, cofactors, ions, and key water molecules can now be docked simultaneously. Interface-residue identities can now be redesigned during docking. By separating low- and high-resolution docking, a study can be optimized for high-throughput virtual screening. The following script is designed to replicate the protocol described by Davis and Baker[26]:

<ROSETTASCRIPTS>

<SCOREFXNS>

<ligand_soft_rep weights = ligand_soft_rep> use a soft potential from the Rosetta database

<Reweight scoretype = hack_elec weight = 0.42/> change the Coloumb electrostatic weight to 0.42

</ligand_soft_rep>

<hard_rep weights = ligand>

<Reweight scoretype = hack_elec weight = 0.42/>

</hard_rep>

</SCOREFXNS>

<LIGAND_AREAS>

<docking_sidechain chain = X cutoff = 6.0 add_nbr_radius = true all_atom_mode = true minimize_ligand = 10/>

<final_sidechain chain = X cutoff = 6.0 add_nbr_radius = true all_atom_mode = true/>

<final_backbone chain = X cutoff = 7.0 add_nbr_radius = false all_atom_mode = true Calpha_restraints = 0.3/>

</LIGAND_AREAS>

<INTERFACE_BUILDERS>

<side_chain_for_docking ligand_areas = docking_sidechain/>

<side_chain_for_final ligand_areas = final_sidechain/>

<backbone ligand_areas = final_backbone extension_window = 3/>

</INTERFACE_BUILDERS>

<MOVEMAP_BUILDERS>

<docking sc_interface = side_chain_for_docking minimize_water = true/>

<final sc_interface = side_chain_for_final bb_interface = backbone minimize_water = true/>

</MOVEMAP_BUILDERS>

<MOVERS>

single movers

<StartFrom name = start_from chain = X>

<Coordinates x = -1.731 y = 32.589 z = -5.039/>

</StartFrom>

<Translate name = translate chain = X distribution = uniform angstroms = 0.01 cycles = 50/>

<Rotate name = rotate chain = X distribution = uniform degrees = 360 cycles = 1000/>

<SlideTogether name = slide_together chain = X/>

<HighResDocker name = high_res_docker chains = X cycles = 6 repack_every_Nth = 3 scorefxn = ligand_soft_rep movemap_builder = docking/>

<FinalMinimizer name = final scorefxn = hard_rep movemap_builder = final/>

<InterfaceScoreCalculator name = add_scores chains = X scorefxn = hard_rep native="inputs/7cpa_7cpa_native.pdb"/>

compound movers

<ParsedProtocol name = low_res_dock>

<Add mover_name = start_from/>

<Add mover_name = translate/>

<Add mover_name = rotate/>

<Add mover_name = slide_together/>

</ParsedProtocol>

<ParsedProtocol name = high_res_dock>

<Add mover_name = high_res_docker/>

<Add mover_name = final/>

</ParsedProtocol>

</MOVERS>

<PROTOCOLS>

<Add mover_name = low_res_dock/>

<Add mover_name = high_res_dock/>

<Add mover_name = add_scores/>

</PROTOCOLS>

</ROSETTASCRIPTS>

The StartFrom Mover moves a small molecule to a specified XYZ-coordinate and is used to position the ligand in proximity to a putative binding site. Translate randomly moves a specified distance in any direction. This is repeated a specified number of times until the ligand does not sterically clash with the protein. Rotate randomly reorients the ligand a specified number of times, looking for a rotation that leads to van der Waals attractive and repulsive scores that pass a threshold. After initial placement of the ligand, SlideTogether moves the ligand toward the protein until the two collide and then backs up the ligand slightly, to ensure that contact between the partners is maintained. The HighResDocker performs cycles of rotamer trials or repacking, coupled with small perturbations of the ligand. FinalMinimizer performs gradient-based minimization of the final docked pose. Finally the InterfaceScoreCalculator records the value of each score term with the docked ligand and after removing the docked ligand. The differences between paired terms represents the interface score. It also calculates the distance the ligand traveled and the ligand radius of gyration. If the structure is known (in case of benchmark studies), root-mean-square distances can be calculated.

Several other ligand-docking specific XML elements are used by the Movers above. LIGAND_AREAS describe parameters specific to each ligand in a multi-ligand docking study. A cutoff distance specified in Ångstroms determines how far away an amino acid residue can be from the ligand and still be considered part of an interface. The neighbor_radius parameter is specified in the ligand-params file and can be added to the specified cutoff distance. All-atom mode checks the distance between each residue and every ligand atom. Otherwise the distance is checked only from the ligand centroid. During high-resolution docking, modest sampling of ligand translation and rotation are coupled to cycles of rotamer trials or repacking. These values can be controlled by the high_res_angstrom and high_res_degrees values, respectively. LIGAND_AREAS specify the degree of ligand flexibility and backbone flexibility around each ligand. Ligand minimization can be turned on by specifying a minimize_ligand value greater than 0. This value represents the size of one standard deviation of ligand torsion-angle rotation (in degrees). By setting Calpha_restraints greater than 0, backbone flexibility is enabled. This value represents the size of one standard deviation of Calpha movement in Ångstroms. INTERFACE_BUILDERS describe how to choose residues that will be part of a protein-ligand interface. The user provides a list of ligand_area names in comma separated form. MOVEMAP_BUILDERS construct descriptions of the degrees of freedom allowed in minimization.

#### Design of DNA-binding proteins

RosettaScripts can be used to perform modeling and design of protein-DNA interfaces. Protein-DNA complexes were first modeled using Rosetta by Havranek et al. [27]. Predictions are made using atom properties and energy forcefield parameters that are based on the default Rosetta energy function[28]. This has subsequently been applied to research into the design of novel specificity for DNA-binding proteins, including the design of individual changes in the nucleotide specificity of homing endonucleases[29], as well as methods to theoretically maximize specificity[28], introduce small protein backbone shifts[30], and optimize clusters of amino acids for changes in multiple adjacent base pairs[28]. The RosettaScripts components can be used to make similar kinds of predictions, as well as to efficiently build, extend, and test new modeling and design protocols. The following protocol redesigns a protein around a DNA molecule, using the multistate-design framework to take into account binding specificity and backbone remodeling.

<ROSETTASCRIPTS>

<TASKOPERATIONS>

<InitializeFromCommandline name = IFC/>

<IncludeCurrent name = IC/>

<RestrictDesignToProteinDNAInterface name = DnaInt base_only = 1 z_cutoff = 3.0 dna_defs = C.-10.GUA/>

<OperateOnCertainResidues name = AUTOprot>

<AddBehaviorRLT behavior = AUTO/>

<ResidueHasProperty property = PROTEIN/>

</OperateOnCertainResidues>

<OperateOnCertainResidues name = ProtNoDes>

<RestrictToRepackingRLT/>

<ResidueHasProperty property = PROTEIN/>

</OperateOnCertainResidues>

<OperateOnCertainResidues name = DnaNoPack>

<PreventRepackingRLT/>

<ResidueHasProperty property = DNA/>

</OperateOnCertainResidues>

</TASKOPERATIONS>

<SCOREFXNS>

<DNA weights = dna/>

</SCOREFXNS>

<FILTERS>

<FalseFilter name = falsefilter/>

</FILTERS>

<MOVERS>

<DnaInterfaceMultiStateDesign name = msd scorefxn = DNA task_operations = IFC,IC,AUTOprot,DnaInt pop_size = 20 num_packs = 1 numresults = 0 boltz_temp = 2 anchor_offset = 15 mutate_rate = 0.8 generations = 5/>

<DesignProteinBackboneAroundDNA name = bb scorefxn = DNA task_operations = IFC,IC,AUTOprot,DnaInt type = ccd gapspan = 4 spread = 3 cycles_outer = 3 cycles_inner = 1 temp_initial = 2 temp_final = 0.6/>

<DnaInterfacePacker name = DnaPack scorefxn = DNA task_operations = IFC,IC,AUTOprot,ProtNoDes,DnaInt binding = 1 probe_specificity = 1/>

<ParsedProtocol name = bb_msd>

<Add mover_name = msd/>

<Add mover_name = bb/>

<Add mover_name = msd/>

</ParsedProtocol>

<LoopOver name = iterbb mover_name = bb_msd filter_name = falsefilter iterations = 1/>

</MOVERS>

<PROTOCOLS>

<Add mover_name = iterbb/>

<Add mover_name = DnaPack/>

</PROTOCOLS>

</ROSETTASCRIPTS>

The DnaInterfacePacker Mover performs side-chain optimization and design of protein-DNA interactions, as well as specificity prediction. DnaInterfaceMultiStateDesign employs a Packer-based genetic algorithm to optimize amino acid sequences to maximize the energy discrimination between the target and alternative DNA sequences, and DesignProteinBackboneAroundDNA introduces small local changes in protein backbone conformation in the vicinity of DNA. The TaskOperation RestrictDesignToProteinDNAInterface automatically limits the freedom of amino acid torsions and mutations to the relevant vicinity of the protein-DNA interface.

## Supporting Information

Supplemental Information S1 **Protocols, input, and output files for all examples given in the paper.**
(BZ2)
